# Rodent research of attention-deficit/hyperactivity disorder: insights into widely used animal models

**DOI:** 10.1186/s42826-025-00255-5

**Published:** 2025-09-23

**Authors:** Juan Carlos Corona

**Affiliations:** Laboratory of Neurosciences, Hospital Infantil de México Federico Gómez, Mexico City, 06720 Mexico

**Keywords:** Attention-deficit/hyperactivity disorder, Dopamine, Animal therapies, Rodent models, Validity

## Abstract

Numerous rodent research models of attention-deficit/hyperactivity disorder (ADHD) have been proposed, including pharmacological, environmental, and genetically generated models. A rodent model for studying ADHD should demonstrate similarities to the disorder by mimicking its three core symptoms (face validity), should align with a theoretically justified pathophysiological basis (construct validity), and should provide insights into unknown aspects of ADHD neurobiology while offering potential new treatments (predictive validity). This review provides an overview of rodent research models, which vary in their pathophysiological alterations, ability to replicate behavioural symptoms, and response to pharmacological treatments. Given this heterogeneity, it remains challenging to determine which rodent model best represents ADHD or its different subtypes. Consequently, validating these models against contemporary medication therapies and testing candidate natural compounds as potential adjuvant treatments is essential. Additionally, combining models induced by neurotoxins, environmental substances, and genetic modifications may help evaluate potential interactions and their impact on ADHD development.

**Supplementary Information:**

The online version contains supplementary material available at 10.1186/s42826-025-00255-5.

## Background

Inattentiveness, hyperactivity, and impulsivity are the key characteristics and clinical symptoms of attention-deficit/hyperactivity disorder (ADHD), the most common neuropsychiatric disorder, with 8% to 12% of children in the United States diagnosed [1–4]. Currently, ADHD has a global prevalence of 8.0% in children and adolescents, while its prevalence in adults is 2.5% [5]. In addition, children and adolescents with ADHD face an increased risk of multimorbidity, as well as a range of behavioural, psychiatric (e.g., depression, drug abuse, and delinquency), and somatic (e.g., asthma and obesity) health issues [6–9]. The standard treatment for ADHD is pharmacotherapy, although psychotherapy, behavioural training, and psychoeducational interventions are also commonly used. The most effective approach often involves a combination of these treatments [2, 10–12]. Currently, ADHD pharmacotherapy consists of psychostimulants such as methylphenidate (MPH) and non-psychostimulants like atomoxetine (ATX), as well as other treatments involving glutamatergic agents [13].

Several studies have provided evidence of abnormalities in dopaminergic neurotransmission in ADHD [14–16], suggesting that these abnormalities are associated with the disorder’s pathophysiology [17–19]. Furthermore, research using animal models of ADHD has also demonstrated abnormalities in dopaminergic neurotransmission [20–22]. In addition, various findings suggest that oxidative stress and neuroinflammation are key pathophysiological factors contributing to the development of ADHD [1, 23].

Increasingly more genes are being identified as involved in the aetiology of ADHD, reinforcing that it is a highly heritable disorder, with an estimated heritability of approximately 76% [24, 25]. ADHD is highly polygenic, with approximately 7,000 genetic variants potentially explaining 90% of single-nucleotide polymorphism heritability, and common genetic variants associated with ADHD have been linked to impairments in attention and verbal reasoning [26]. Exposure to various environmental factors—such as pesticides, prematurity or low birth weight, maternal alcohol and tobacco use, maternal stress, nutritional deficiencies, obesity during pregnancy, and viral infections—has also been identified as contributing risk factors for ADHD [27–29].

## Main text

### Animal models

**Fig. 1:**
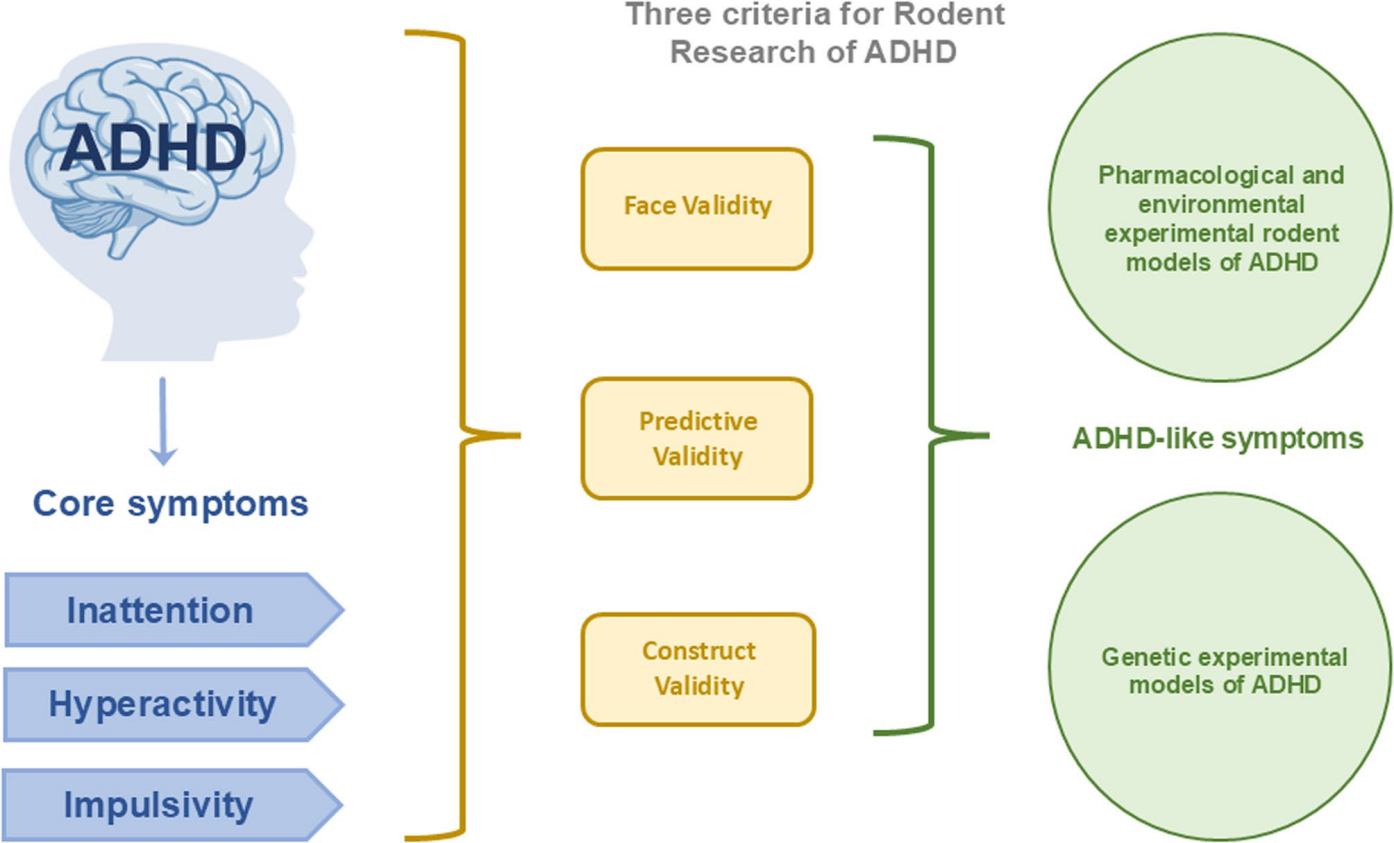
Core symptoms of ADHD and criteria for animal models. This figure summarises human ADHD alongside the three validation criteria—predictive, face, and construct validity—used to evaluate pharmacological, environmental, and genetic rodent models of the disorder. These models are valuable for studying specific molecular, genetic, and cellular mechanism associated with ADHD

Rodents are widely used as experimental animal models to study various diseases, including diabetes, neurodegenerative disorders, and neuropsychiatric conditions [30–34], giving insights into the biomedical and evolutionary mechanisms of the nervous system, disease, and behaviour. Rodent models contribute to our understanding of the neuropathological, neurochemical, molecular, cellular, genetic, and environmental aspects observed in ADHD. These models of ADHD offer several advantages: they are less expensive to maintain, permit hypothesis testing by allowing control over genetic and environmental variables, enable the use of invasive methods not possible in humans, and provide better control over factors such as diet. Additionally, their physiology is relatively similar to that of humans (Fig. 1). These models can be useful for studying, the effects of various natural compounds or medications on different brain areas or specific tissues [35–37]. However, it should be noted that while rodent models can capture certain aspects of the human disorder, they do not always reflect the full heterogeneity of ADHD and do not fully account for the symptom variability observed across individual patients. Although rodents have simpler nervous systems, their basic behavioural mechanisms are similar to those of humans, and while it may be difficult, it is not impossible to study some complex cognitive behaviours [21]. Rodents do not exhibit human-like language, but certain features of language can be studied in rats—particularly their ability to recognise and generalise rule-based auditory patterns. This suggest that rats can detect abstract structural patterns in sound sequences in a way that is similar to how humans process syntax in language [38]. Flexible decision-making in complex environments is a hallmark of intelligent behaviour, and using a combination of behavioural, computational, and electrophysiological methods, it has been shown that both rats and humans share conserved mechanisms of cognitive flexibility in such environments [39]. A deeper understanding of the translational value of rodent models—due to their similarities with human physiology and disease mechanisms—can help researchers advance insights that support the development of targeted therapies for neuropsychiatric disorders.

### Model criteria

**Fig. 2:**
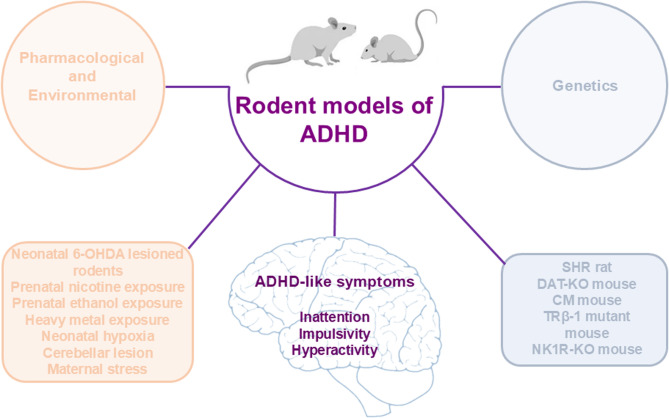
Rodent research models of ADHD and their characteristic features. This figure summarises the various experimental rodent models of ADHD, including those induced with different substances (pharmacological and environmental) and genetic models. Each model highlights key behavioural and neurobiological characteristics relevant to ADHD. Further details are provided in the text

A suitable rodent research model of ADHD must meet three criteria (Fig. 1). (1) the model must replicate the three core symptoms of the human disorder: inattention, hyperactivity, and impulsivity (face validity). Although reproducing core symptoms can enhance face validity, it is not a strict requirement for a model to be considered valid. (2) the model must align with a theoretically justified pathophysiological basis of the disorder (construct validity). Construct validity has not been achieved for ADHD because the aetiology of the disorder remains unknown. Even so, partial construct validity has been demonstrated through insights into some underlying mechanisms. (3) the model should predict novel or future aspects of neurobiology, genetics, treatment responses, and ADHD-related behaviour (predictive validity). Several comprehensive review articles discuss the validity criteria of various rodent models of ADHD [22, 35–37, 40–42]. Although construct validity is often regarded as the key form of validity, it is not an absolute requirement for a model to be considered valid or suitable. Some models rely primarily on face and/or predictive validity but still provide valuable insights into the disorder. This presents a challenge in many neuropsychiatric disorders, including ADHD, where neurobiology is not yet fully understood. ADHD is highly heterogeneous and has a multi-aetiological basis, making construct validity difficult to achieve. Consequently, many of the currently available models demonstrate at least some degree of face, construct, and predictive validity for one or more ADHD symptom subgroups. Rodent research models of ADHD are primarily classified into two categories: those induced using various substances (pharmacological and environmental) and those that are genetically induced. The most widely used rodent research models for ADHD are summarised in Fig. 2.

### Pharmacological and environmental experimental rodent models of ADHD

#### Neonatal 6-hydroxydopamine-lesioned rodents

6-Hydroxydopamine (6-OHDA) has high affinity for the dopamine transporter (DAT) and norepinephrine transporter, allowing it to accumulate in both dopaminergic and noradrenergic neurons. Once inside the cell, 6-OHDA undergoes auto-oxidation, leading to the formation of reactive oxygen species. Additionally, 6-OHDA can inhibit mitochondrial complex I, generating hydrogen peroxide, superoxide, and hydroxyl radicals, ultimately resulting in adenosine triphosphate depletion [43–45]. The destruction of dopaminergic projections in the brains of neonatal 6-OHDA-lesioned rodents (rats and mice) leads to hyperactivity, impaired spatial discrimination learning, and changes in attention [46–53]. Impulsive-like behaviour has been observed in adult mice [47], and later studies demonstrated that 6-OHDA-lesioned mice also exhibited impulsivity-like behaviour during adolescence [46]. 6-OHDA-lesioned rodents, in combination with desipramine (to preserve noradrenergic neurons), represent the most widely used neurotoxin-based experimental model for studying ADHD. Many of the observed behavioural deficits are linked to acute adaptive alterations in the dopaminergic system caused by 6-OHDA lesions [22, 47]. These rodents exhibit decreased dopamine levels, reduced striatal DAT density, increased dopamine receptor D4 expression, and alterations in serotonin receptors [48, 50, 54–56]. Their behavioural deficits can be improved with treatment using psychostimulants or ATX [47–49, 57–59]. To evaluate the effects and mechanisms of neonatal 6-OHDA-lesioned rodents as an experimental ADHD model, three routes of administration have been used: intracisternal injection [57, 59], injection into the lateral ventricle [46, 47], and unilateral intrastriatal injection [52, 53, 60, 61]. Intrastriatal lesions can lead to the progressive loss of dopaminergic neurons in the substantia nigra pars compacta, resulting in dopamine depletion [53, 60, 61]. However, the route of 6-OHDA administration may influence outcomes and treatment responses. These rodent models demonstrate predictive validity, as psychostimulant and ATX treatments reduce hyperactivity and attention deficits. Construct validity is supported by changes in the dopaminergic and serotonergic systems, while face validity is established by the observed behavioural deficits observed. Thus, this model could serve as a reliable tool for studying ADHD. The characteristic features of pharmacological and environmental experimental models of ADHD are summarised in Table 1.

**Table 1:** Features of pharmacological and environmental experimental rodent models of ADHD

Model	Face Validity	Predictive Validity	Construct Validity	References
Neonatal 6-OHDA lesioned rodents	Inattention Hyperactivity Impulsivity	Hyperactivity and inattention improved by amphetamines, MPH, and ATX	Dopaminergic and serotonergic modifications	[47, 53, 59]
Prenatal nicotine exposure	Inattention Hyperactivity Impulsivity	Hyperactivity improved by MPH	Dopaminergic modifications	[62, 63]
Prenatal ethanol exposure	Inattention Hyperactivity Impulsivity	Dopaminergic modifications improved by MPH and amphetamines	Dopaminergic modifications	[64, 65]
Heavy metal exposure	Hyperactivity No data regarding inattention No data regarding impulsivity	Hyperactivity improved by amphetamines and MPH	Dopaminergic modifications	[66, 67]
Neonatal hypoxia	Hyperactivity No data regarding inattention No data regarding impulsivity	Hyperactivity improved by amphetamines	Catecholaminergic and serotonergic modifications	[68]
Cerebellar lesion	Hyperactivity No data regarding inattention No data regarding impulsivity	No predictive validity data	No data regarding modifications of catecholaminergic system	[69, 70]
Maternal stress	Hyperactivity Inattention Impulsivity	Hyperactivity improved by dopamine receptor antagonist	Dopaminergic modifications	[71, 72]

### Prenatal nicotine exposure

Exposure to nicotine during the prenatal and postnatal stages is considered an environmental risk factor for ADHD. Children born to mothers who smoked cigarettes before, during, or immediately after pregnancy have a two-fold higher risk of developing ADHD [73, 74]. A mouse model of prenatal nicotine exposure via drinking water exhibited hyperactivity, decreased dopamine turnover in the frontal cortex, reduced cortical volume, and sensitivity to oral MPH treatment [62]. Similarly, adolescent rats prenatally exposed to nicotine also displayed hyperactivity [75]. In another study, nicotine administered to pregnant mice through drinking water induced hyperactivity that was transmitted across generations, although only the founder generation was directly exposed. Notably, this transmission occurred exclusively via the maternal lineage [76]. Prenatal nicotine exposure through drinking water has also been shown to produce impulsive behaviours in rats and disrupt neural activity in the medial prefrontal cortex [63]. Additionally, in mice, nicotine exposure through drinking water resulted in significant deficits in attention and working memory in male offspring, but not in females [77]. However, because nicotine was administered via drinking water rather than through smoking boxes, this model could be considered more of a pharmacological rather than an environmental model. Variations in behavioural findings from these animal models are likely due to differences in routes of administration, doses, species, and timing of exposure. Despite this variability, prenatal nicotine exposure models have demonstrated face validity (behavioural deficits observed), construct validity (dopaminergic modifications), and predictive validity (hyperactivity improved by MPH treatment) in relation to ADHD.

#### Prenatal ethanol exposure

Drinking alcohol at any stage of pregnancy has been associated with an increased risk of ADHD in infants [78, 79]. Prenatal ethanol exposure in rats leads to neurochemical and morphological deficits in the brain, resulting in postnatal hyperactivity, impulsivity, and attention deficits [64, 65, 80]. Rats prenatally exposed to ethanol exhibit dysregulation of dopaminergic neurons in the ventral tegmental area, a dysfunction that can be regulated by MPH and amphetamines [81–84]. These findings support the presence of predictive validity (response to MPH and amphetamines), face validity (inattention, hyperactivity and impulsivity), and construct validity (dopaminergic modifications) in this model. While prenatal ethanol exposure is a recognised risk factor for ADHD, behavioural findings from animal model remain varied. Further research is needed to fully validate prenatal ethanol exposure as a reliable model for ADHD.

#### Heavy metal exposure

Excessive exposure to heavy metals is detrimental to neurodevelopmental processes, exerting neurotoxic effects that can impair cognitive functions. This has led to the implication of heavy metal exposure in susceptibility to ADHD [85]. Lead exposure during early development has been shown to induce hyperactivity in mice, an effect that was prevented with MPH and amphetamine treatment [66, 86, 87]. In rats chronically exposed to lead, dopamine turnover was found to be decreased in the striatum and nucleus accumbens [88]. Similarly, exposure to cadmium and manganese produced hyperactivity, spatial learning, and memory deficits in rats, effects that were associated with altered dopamine receptor and DAT levels [67, 89, 90]. While these experimental models only demonstrate elements of predictive validity (hyperactivity prevented by MPH and amphetamines), face validity (hyperactivity), and construct validity (dopaminergic modifications), further research is needed to fully establish their relevance as models of ADHD.

#### Neonatal hypoxia

Prenatal and perinatal brain hypoxia leads to behavioural and neurochemical changes, increasing the neurodevelopmental risk for ADHD [91]. Hypoxia induced by nitrogen in postnatal rats (90%–100% nitrogen exposure within 24 h of birth) has been shown to mimic the hyperactivity and learning impairments observed in ADHD [68, 92, 93]. These effects have been studied between postnatal days 2 and 10 [68, 94–97]. However, by 6–9 weeks of age, the hyperactivity was found to normalise [92, 94, 98], a characteristic observed in many individuals with ADHD after the teenage years. The hyperactivity was also alleviated with psychostimulant treatment [92]. Hypoxia induces age-dependent adaptive monoaminergic alterations. In the acute phase, there is a decrease in dopamine and norepinephrine levels in the cortex and striatum, while serotonin metabolite 5-hydroxyindoleacetic acid is increased in both the cerebellum and cortex [99]. Face validity is supported by the presence of hyperactivity, although further research is needed to evaluate attention deficits and impulsivity. Construct validity is linked to alterations in the catecholaminergic and serotonergic systems, while predictive validity is supported by the observed effect of amphetamines in reducing hyperactivity. Despite these findings, further research is required to fully determine the validity of hypoxia-induced models for ADHD.

#### Cerebellar lesions

The cerebellum has been found to be smaller in patients with ADHD, suggesting its involvement in the disorder’s development [100, 101]. While the cerebellum is primarily known for its role in coordination and motor learning, it is also involved in attentional control, working memory, emotion regulation, action planning, and response timing by processing input from various brain regions, the spinal cord, and sensory receptors [102]. Lesions to the cerebellum—induced using substances such as methylazoxymethanol, alpha-difluoromethylornithine, and dexamethasone—within 5–12 days post-birth result in cerebellar growth inhibition and the development of hyperactivity in rats [69, 70, 103, 104]. However, amphetamine treatment increased hyperactivity rather than alleviating it [103]. Face validity is partially supported because the lesions induce hyperactivity. Predictive validity is absent, given that amphetamine treatment exacerbates rather than reduces hyperactivity. Construct validity is difficult to determine due the lack of data on alterations in the catecholaminergic system in this model. Although current findings are insufficient to validate cerebellar lesions in rats as an ADHD model, this approach may provide valuable insights into the role of the cerebellum in ADHD pathophysiology. Further research is needed to establish its relevance as an experimental model.

#### Maternal stress

There is some evidence that prenatal maternal stress may be a risk factor for ADHD [105]. In maternally stressed mice, adult offspring exhibited hyperactivity and alterations in the midbrain dopaminergic system; however, administration of a dopamine receptor antagonist reduced hyperactivity [71]. Additionally, maternal stress-induced ADHD-like behavioural phenotypes in mouse offspring were linked to changes in plasma gamma aminobutyric acid (GABA) metabolism and dopamine concentration [106]. Cortisol is a key biological factor associated with stress-induced psychiatric disorders. Prenatal exposure to high corticosterone has been shown to induce ADHD-like behaviours—including impulsivity, hyperactivity, and inattention—as well as cognitive deficits such as learning and memory impairments. These effects are mediated through developmental delays in hippocampal CA1 neurons in rats [72, 107]. This experimental model demonstrates elements of face validity (inattention, hyperactivity, and impulsivity), predictive validity (hyperactivity reduced by dopamine receptor antagonists), and construct validity (dopaminergic modifications). However, further research is needed to fully establish its reliability as an ADHD model.

### Genetic experimental models of ADHD

#### The spontaneously hypertensive rat

The spontaneously hypertensive rat (SHR) is the most extensively studied genetic rat model of ADHD. Developed in the 1960 s [108] through inbreeding of the normotensive Wistar–Kyoto strain, the SHR was originally bred for hypertension, although hypertension has not been reported as a characteristic of ADHD in humans. Despite this, SHRs exhibit high spontaneous motor activity and display all of the core behavioural characteristics of ADHD, including impulsivity, hyperactivity, poor sustained attention, and poor stability of performance when compared with Wistar–Kyoto rats [109–112]. Thus, face validity is supported by behavioural similarities to ADHD, construct validity is reinforced by alterations in the catecholaminergic system, and predictive validity is demonstrated by the efficacy of monoaminergic drugs in reducing ADHD-like behaviours [109, 113, 114]. However, the presence of hypertension in SHRs cannot be overlooked because increased blood pressure may influence behaviour, potentially confounding ADHD-related findings. A recent study also suggests that SHRs might not be a suitable model for the inattentive or combined subtypes of ADHD [115]. Face validity is supported by hyperactivity, impulsivity, and poor attention; the predictive validity is supported by the improvement with monoaminergic drugs; and construct validity is supported by data on catecholaminergic system alterations. Additional research is needed to determine whether behavioural deficits in SHRs are a direct reflection of ADHD-like pathology or a secondary effect of hypertension-induced brain alterations. Further investigation is also needed to explore the role of the SHRs as an ADHD model through alternative approaches. The characteristic features of genetic experimental models of ADHD are summarised in Table 2.

**Table 2:** Features of genetic experimental rodent models of ADHD

Model	Face Validity	Predictive Validity	Construct Validity	References
SHR rat	Poor attention Hyperactivity Impulsivity	Symptoms improved by monoaminergic drugs	Catecholaminergic modifications	[108, 110, 111]
DAT-KO mouse	Inattention Hyperactivity Impulsivity	Symptoms improved by MPH	Dopaminergic modifications	[116, 117]
CM mouse	Inattention Hyperactivity Impulsivity	Hyperactivity improved by amphetamines	Catecholaminergic modifications	[118–120]
TRβ−1 mutant mouse	Inattention Hyperactivity Impulsivity	Hyperactivity improved by MPH	Dopaminergic modifications	[121, 122]
NK1R-KO mouse	Inattention Hyperactivity Impulsivity	Hyperactivity improved by amphetamines and MPH Impulsivity improved by ATX	Catecholaminergic and serotonergic modifications	[123, 124]

### The DAT knockout mouse

The DAT knockout (DAT-KO) mouse is one of the most extensively characterised transgenic experimental models for ADHD. This model lacks the DAT due to deletion of the *Slc6a3* gene, which encodes the DAT protein responsible for dopamine reuptake into presynaptic terminals [125–127]. DAT-KO mice exhibit ADHD-like symptoms along with alterations in spatial memory [116, 117]. MPH treatment ameliorates their hyperactive, inattentive, and impulsive-like behaviours [116]. The hyperactivity observed in DAT-KO mice is linked to a significant reduction in dopamine clearance [128]. As a result, extracellular dopamine levels in the brain are increased approximately five-fold due to slow dopamine clearance [125]. Face validity is supported by behavioural similarities to ADHD, construct validity is reinforced by alterations in the catecholaminergic system, and predictive validity is demonstrated by the efficacy of MPH in reducing ADHD-like behaviours. However, findings regarding DAT levels in patients with ADHD are inconsistent. Some studies have revealed increased DAT levels in the striatum of both children and adults with ADHD [56, 129, 130], whereas others have found reduced DAT expression in patients with ADHD using brain imaging studies [131]. To fully validate the DAT-KO model, further research is needed to clarify the role of DAT in ADHD pathophysiology and resolve these discrepancies.

#### The coloboma mutant mouse

The coloboma mutant (CM) mouse was developed through neutron irradiation, which induced a mutation on chromosome 2, disrupting several genes, including synaptosomal-associated protein 25 kDa (SNAP-25). The behavioural deficits observed in CM mice are associated with SNAP-25 dysfunction [120, 132, 133]. SNAP-25 is a crucial protein for neurotransmitter release because it facilitates the fusion of neurotransmitter vesicles with the presynaptic membrane. Due to this mutation, dopamine release in the striatum of CM mice is almost entirely absent [118]. CM mice exhibit behavioural deficits including impulsivity, spontaneous hyperactivity, impaired inhibition in a delayed reinforcement task, and delayed neurodevelopment [119, 120, 133]. Construct validity is supported by alterations in catecholaminergic systems—specifically, an increase in the noradrenergic activity and a decrease in the dopaminergic function. Face validity is assumed based on the presence of behavioural deficits resembling ADHD symptoms. Predictive validity is suggested by the effectiveness of psychostimulants in modulating ADHD-like behaviours. These characteristics indicate that the CM mouse may serve as a useful genetic model for studying ADHD; however, further research is needed to fully validate its applicability.

#### The thyroid receptor β−1 mutant mouse

The thyroid receptor β−1 (TRβ−1) mutant mouse is an animal model used to study ADHD. This mouse carries a mutant human thyroid hormone receptor β gene, derived from a patient diagnosed with resistance to thyroid hormone [121]. The disease is heritable and characterised by elevated levels of thyroid hormone and thyroxine, sometimes with elevated thyroid-stimulating hormone, tachycardia, and hearing loss [134]. Approximately 70% of children with this condition are diagnosed with ADHD [135, 136]. The TRβ−1 mutant mouse exhibits impulsivity, hyperactivity, and inattention, with elevated thyroid-stimulating hormone levels observed at 33 days of age. The behavioural deficits persist into adulthood [121, 122, 137]. Moreover, these deficits are related to the catecholaminergic system, as the mice show sensitivity to MPH treatment and display elevated dopamine turnover [121]. Nevertheless, the role of the thyroid system in ADHD remains unclear, suggesting that modifications in thyroid function could lead to alterations in brain development, resulting in ADHD-like behavioural phenotypes [138]. The TRβ−1 mutant mouse demonstrates face validity, as it exhibits all three core symptoms of ADHD. Predictive validity is supported by sensitivity to MPH treatment, while construct validity is indicated by alterations in the catecholaminergic system and evidence of developmental disturbances. While this model provides insights into the potential involvement of the thyroid system in ADHD, further studies are needed to establish its validity more conclusively.

#### The neurokinin-1 receptor knockout mouse

The neurokinin-1 receptor knockout (NK1R-KO) mouse, also known as the tachykinin receptor-1 knockout mouse, has been proposed as an experimental model of ADHD [124, 139, 140]. NK1 receptors are G-protein-coupled receptors activated by the binding of substance P and are expressed in the brain [124]. As a tachykinin neuropeptide, substance P is localised in brain regions involved in cognitive performance, motor control, and mood regulation. The NK1R-KO mouse exhibits locomotor hyperactivity in various experimental environments [123, 141, 142]. Later studies demonstrated that this mouse model also displays inattentive and impulsive-like behaviours, further supporting its relevance to ADHD. Given that it exhibits the core behavioural symptoms of the disorder, the NK1R-KO mouse demonstrates strong face validity as an animal model. The behavioural abnormalities in NK1R-KO mice are ameliorated by MPH, amphetamines, and ATX, indicating predictive validity [123, 143–146]. Additionally, findings suggest that NK1R-KO mice show alterations in catecholaminergic and serotonergic systems, further supporting construct validity [141, 147].

## Conclusions

The criteria for validating rodent research models of ADHD depend, among other factors, on the behavioural alterations they display, including inattention, hyperactivity, and impulsivity, as well as the pathophysiological basis of the disorder, altered gene expression (given the highly polygenic predisposition to ADHD), and their response to medications. At present, validation of experimental rodent models remains incomplete. However, each model has its own strengths and weaknesses, contributing to a better understanding of different aspects of ADHD. Because many rodent models have not been thoroughly examined for inattention, impulsivity, and cognitive deficits, there is a pressing need for better pharmacological characterisation. Additionally, the genes implicated in ADHD, given its highly polygenic and heterogeneous nature, are often not adequately considered in many experimental rodent models, nor are the different subtypes of ADHD. Genetic models can serve as important tools to study disruptions in specific physiological pathways, such as those seen in certain knockout models, although these disturbances are not necessarily exclusive to ADHD; other disorders may exhibit similar alterations. While face validity and predictive validity can be more readily assessed, construct validity remains difficult to establish. Therefore, careful examination of evidence from experimental animal models, in parallel with clinical studies in humans, is essential. Extensive validation of models that meet these criteria is necessary before forming a solid theory about the aetiology of ADHD, particularly because current knowledge of its neurobiology from human studies remains limited. This limitation arises from ADHD’s extreme heterogeneity, multiple aetiologies, and complex multifactorial phenotype. Although hyperactivity is a key characteristic, it is insufficient on its own to establish adequate face validity. Rodent models that present abnormalities not typically observed in patients with ADHD should also be critically evaluated—for example, the presence of hypertension in SHRs, which is not a defining feature of ADHD. However, it is important to note that SHRs do not develop hypertension until adulthood (10–12 weeks of age), whereas hyperactivity is observed earlier, during adolescence (3–4 weeks of age) [21, 148]. Currently, the validation of experimental models is primarily based on behavioural criteria (face validity), but there remains a poor understanding of ADHD’s construct validity. A key aspect of validation is the model’s responsiveness to ADHD medications (predictive validity), whether psychostimulants or non-psychostimulants. Among all the experimental rodent models discussed, only a few exhibit an adequate response to the psychostimulants commonly used to treat ADHD. Some models respond only to MPH or amphetamines alone, while others fail to respond to either treatment. Additionally, to achieve predictive validity, experimental models of ADHD must also be assessed for their response to ATX, a widely used non-psychostimulant treatment. Therefore, further rigorous research is required to validate these models, ensuring that some can be assessed not only against current drug therapies but also in relation to potential alternative or co-adjuvant therapies. In summary, research models that demonstrate both face validity and predictive validity could be valuable for investigating contemporary ADHD drug therapies and testing candidate compounds suitable for adjuvant treatment. Future studies should focus on experimental animal models that capture the heterogeneous and multifactorial phenotype of ADHD. Moreover, genetic models, as well as those induced by neurotoxins or environmental substances, should be combined to evaluate potential interactions and assess their impact on ADHD development.

## Supplementary Information

Supplementary Material 1.

## Data Availability

All data presented in the manuscript were collected through a literature search.

## Abbreviations

6-OHDA: 6-hydroxydopamine
ADHD: attention-deficit/hyperactivity disorder
ATX: atomoxetine
CM: coloboma mutant
DAT: dopamine transporter
DAT-KO: dopamine transporter knockout
MPH: methylphenidate
NK1R-KO: neurokinin-1 receptor knockout
SHR: spontaneously hypertensive rat
SNAP-25: synaptosomal-associated protein 25 kDa
TRβ-1: thyroid receptor β-1

## References

[1] Corona JC. Role of oxidative stress and neuroinflammation in attention-deficit/hyperactivity disorder. Antioxidants. 2020;9(11):1039. 33114154 10.3390/antiox9111039 PMC7690797

[2] Posner J, Polanczyk GV, Sonuga-Barke E. Attention-deficit hyperactivity disorder. Lancet. 2020;395(10222):450–62. 31982036 10.1016/S0140-6736(19)33004-1 PMC7880081

[3] Koirala S, Grimsrud G, Mooney MA, Larsen B, Feczko E, Elison JT, et al. Neurobiology of attention-deficit hyperactivity disorder: historical challenges and emerging frontiers. Nat Rev Neurosci. 2024;25(12):759–75. 39448818 10.1038/s41583-024-00869-z PMC13404203

[4] Danielson ML, Bitsko RH, Ghandour RM, Holbrook JR, Kogan MD, Blumberg SJ. Prevalence of parent-reported ADHD diagnosis and associated treatment among U.S. children and adolescents, 2016. J Clin Child Adolesc Psychol. 2018;47(2):199–212. 29363986 10.1080/15374416.2017.1417860 PMC5834391

[5] Ayano G, Demelash S, Gizachew Y, Tsegay L, Alati R. The global prevalence of attention deficit hyperactivity disorder in children and adolescents: an umbrella review of meta-analyses. J Affect Disord. 2023;339:860–6. 37495084 10.1016/j.jad.2023.07.071

[6] Cortese S, Tessari L. Attention-deficit/hyperactivity disorder (ADHD) and obesity: update 2016. Curr Psychiatry Rep. 2017;19(1):4. 28102515 10.1007/s11920-017-0754-1 PMC5247534

[7] Instanes JT, Klungsoyr K, Halmoy A, Fasmer OB, Haavik J. Adult ADHD and comorbid somatic disease: a systematic literature review. J Atten Disord. 2018;22(3):203–28. 27664125 10.1177/1087054716669589 PMC5987989

[8] Fredriksen M, Dahl AA, Martinsen EW, Klungsoyr O, Faraone SV, Peleikis DE. Childhood and persistent ADHD symptoms associated with educational failure and long-term occupational disability in adult ADHD. Atten Defic Hyperact Disord. 2014;6(2):87–99. 24497125 10.1007/s12402-014-0126-1 PMC4033786

[9] Wolraich ML, Hagan JF Jr., Allan C, Chan E, Davison D, Earls M, et al. Clinical practice guideline for the diagnosis, evaluation, and treatment of Attention-Deficit/Hyperactivity disorder in children and adolescents. Pediatrics. 2019;144(4):e20192528. 31570648 10.1542/peds.2019-2528 PMC7067282

[10] Briars L, Todd T. A review of pharmacological management of Attention-Deficit/Hyperactivity disorder. J Pediatr Pharmacol Ther. 2016;21(3):192–206. 27453697 10.5863/1551-6776-21.3.192 PMC4956327

[11] Brown KA, Samuel S, Patel DR. Pharmacologic management of attention deficit hyperactivity disorder in children and adolescents: a review for practitioners. Transl Pediatr. 2018;7(1):36–47. 29441281 10.21037/tp.2017.08.02 PMC5803014

[12] Cortese S. Pharmacologic treatment of attention deficit-hyperactivity disorder. N Engl J Med. 2020;383(11):1050–6. 32905677 10.1056/NEJMra1917069

[13] Corona JC. Pharmacological approaches for the treatment of attention-deficit/hyperactivity disorder. In: Kyser BM, editor. Attention-Deficit hyperactivity disorder: Diagnosis, prevalence and treatment. New York: Nova Science; 2021. pp. 1–39.

[14] Forssberg H, Fernell E, Waters S, Waters N, Tedroff J. Altered pattern of brain dopamine synthesis in male adolescents with attention deficit hyperactivity disorder. Behav Brain Funct. 2006;2:40. 17144907 10.1186/1744-9081-2-40 PMC1698925

[15] Genro JP, Kieling C, Rohde LA, Hutz MH. Attention-deficit/hyperactivity disorder and the dopaminergic hypotheses. Expert Rev Neurother. 2010;10(4):587–601. 20367210 10.1586/ern.10.17

[16] Swanson JM, Kinsbourne M, Nigg J, Lanphear B, Stefanatos GA, Volkow N, et al. Etiologic subtypes of attention-deficit/hyperactivity disorder: brain imaging, molecular genetic and environmental factors and the dopamine hypothesis. Neuropsychol Rev. 2007;17(1):39–59. 17318414 10.1007/s11065-007-9019-9

[17] Corona JC. Natural compounds for the management of parkinson’s disease and Attention-Deficit/Hyperactivity disorder. Biomed Res Int. 2018;2018:4067597. 30596091 10.1155/2018/4067597 PMC6282143

[18] Del Campo N, Chamberlain SR, Sahakian BJ, Robbins TW. The roles of dopamine and noradrenaline in the pathophysiology and treatment of attention-deficit/hyperactivity disorder. Biol Psychiatry. 2011;69(12):e145–57. 21550021 10.1016/j.biopsych.2011.02.036

[19] Prince J. Catecholamine dysfunction in attention-deficit/hyperactivity disorder: an update. J Clin Psychopharmacol. 2008;28(3 Suppl 2):S39–45. 18480676 10.1097/JCP.0b013e318174f92a

[20] Russell V, de Villiers A, Sagvolden T, Lamm M, Taljaard J. Altered dopaminergic function in the prefrontal cortex, nucleus accumbens and caudate-putamen of an animal model of attention-deficit hyperactivity disorder–the spontaneously hypertensive rat. Brain Res. 1995;676(2):343–51. 7614004 10.1016/0006-8993(95)00135-d

[21] Russell VA, Sagvolden T, Johansen EB. Animal models of attention-deficit hyperactivity disorder. Behav Brain Funct. 2005;1:9. 16022733 10.1186/1744-9081-1-9 PMC1180819

[22] Sontag TA, Tucha O, Walitza S, Lange KW. Animal models of attention deficit/hyperactivity disorder (ADHD): a critical review. Atten Defic Hyperact Disord. 2010;2(1):1–20. 21432586 10.1007/s12402-010-0019-x

[23] Vazquez-Gonzalez D, Carreon-Trujillo S, Alvarez-Arellano L, Abarca-Merlin DM, Dominguez-Lopez P, Salazar-Garcia M, et al. A potential role for neuroinflammation in ADHD. Adv Exp Med Biol. 2023;1411:327–56. 36949317 10.1007/978-981-19-7376-5_15

[24] Faraone SV, Perlis RH, Doyle AE, Smoller JW, Goralnick JJ, Holmgren MA, et al. Molecular genetics of attention-deficit/hyperactivity disorder. Biol Psychiatry. 2005;57(11):1313–23. 15950004 10.1016/j.biopsych.2004.11.024

[25] Faraone SV, Larsson H. Genetics of attention deficit hyperactivity disorder. Mol Psychiatry. 2019;24(4):562–75. 29892054 10.1038/s41380-018-0070-0 PMC6477889

[26] Demontis D, Walters GB, Athanasiadis G, Walters R, Therrien K, Farajzadeh L, et al. Genome-wide analyses of ADHD identify 27 risk loci, refine the genetic architecture and implicate several cognitive domains. Nat Genet. 2023;55(2):198–208. 36702997 10.1038/s41588-022-01285-8 PMC10914347

[27] Knopik VS, Sparrow EP, Madden PA, Bucholz KK, Hudziak JJ, Reich W, et al. Contributions of parental alcoholism, prenatal substance exposure, and genetic transmission to child ADHD risk: A female twin study. Psychol Med. 2005;35(5):625–35. 15918339 10.1017/s0033291704004155

[28] Thapar A, Cooper M, Eyre O, Langley K. What have we learnt about the causes of ADHD? J Child Psychol Psychiatry. 2013;54(1):3–16. 22963644 10.1111/j.1469-7610.2012.02611.x PMC3572580

[29] Froehlich TE, Anixt JS, Loe IM, Chirdkiatgumchai V, Kuan L, Gilman RC. Update on environmental risk factors for attention-deficit/hyperactivity disorder. Curr Psychiatry Rep. 2011;13(5):333–44. 21779823 10.1007/s11920-011-0221-3 PMC3277258

[30] Baker M, Hong SI, Kang S, Choi DS. Rodent models for psychiatric disorders: problems and promises. Lab Anim Res. 2020;36:9. 32322555 10.1186/s42826-020-00039-z PMC7161141

[31] Dawson TM, Golde TE, Lagier-Tourenne C. Animal models of neurodegenerative diseases. Nat Neurosci. 2018;21(10):1370–9. 30250265 10.1038/s41593-018-0236-8 PMC6615039

[32] Nestler EJ, Hyman SE. Animal models of neuropsychiatric disorders. Nat Neurosci. 2010;13(10):1161–9. 20877280 10.1038/nn.2647 PMC3750731

[33] Salazar-Garcia M, Corona JC. The use of natural compounds as a strategy to counteract oxidative stress in animal models of diabetes mellitus. Int J Mol Sci. 2021;22(13):7009. 34209800 10.3390/ijms22137009 PMC8268811

[34] Mukherjee P, Roy S, Ghosh D, Nandi SK. Role of animal models in biomedical research: a review. Lab Anim Res. 2022;38(1):18. 35778730 10.1186/s42826-022-00128-1 PMC9247923

[35] Kim D, Yadav D, Song M. An updated review on animal models to study attention-deficit hyperactivity disorder. Transl Psychiatry. 2024;14(1):187. 38605002 10.1038/s41398-024-02893-0 PMC11009407

[36] Regan SL, Williams MT, Vorhees CV. Review of rodent models of attention deficit hyperactivity disorder. Neurosci Biobehav Rev. 2022;132:621–37. 34848247 10.1016/j.neubiorev.2021.11.041 PMC8816876

[37] Kantak KM. Rodent models of attention-deficit hyperactivity disorder: an updated framework for model validation and therapeutic drug discovery. Pharmacol Biochem Behav. 2022;216:173378. 35367465 10.1016/j.pbb.2022.173378

[38] Celma-Miralles A, Toro JM. Discrimination of temporal regularity in rats (*Rattus norvegicus*) and humans (Homo sapiens). J Comp Psychol. 2020;134(1):3–10. 31589060 10.1037/com0000202

[39] Bähner F, Popov T, Boehme N, Hermann S, Merten T, Zingone H, et al. Species-conserved mechanisms of abstract rule learning promote cognitive flexibility in complex environments. BioRxiv. 2024. 10.1101/2022.11.14.516439.

[40] Sagvolden T, Russell VA, Aase H, Johansen EB, Farshbaf M. Rodent models of attention-deficit/hyperactivity disorder. Biol Psychiatry. 2005;57(11):1239–47. 15949994 10.1016/j.biopsych.2005.02.002

[41] Russell VA. Overview of animal models of attention deficit hyperactivity disorder (ADHD). Curr Protoc Neurosci. 2011. 10.1002/0471142301.ns0935s54. 21207367 10.1002/0471142301.ns0935s54

[42] Bari A, Robbins TW. Animal models of ADHD. Curr Top Behav Neurosci. 2011;7:149–85. 21287324 10.1007/7854_2010_102

[43] Heikkila RE, Cohen G. 6-hydroxydopamine: evidence for superoxide radical as an oxidative intermediate. Science. 1973;181(4098):456–7. 4718113 10.1126/science.181.4098.456

[44] Heikkila RE, Cohen G. In vivo generation of hydrogen peroxide from 6-hydroxydopamine. Experientia. 1972;28(10):1197–8. 5087037 10.1007/BF01946168

[45] Heikkila R, Cohen G. Inhibition of biogenic amine uptake by hydrogen peroxide: a mechanism for toxic effects of 6-hydroxydopamine. Science. 1971;172(3989):1257–8. 5576164 10.1126/science.172.3989.1257

[46] Bouchatta O, Manouze H, Ba-M’Hamed S, Landry M, Bennis M. Neonatal 6-OHDA lesion model in mouse induces cognitive dysfunctions of attention-deficit/hyperactivity disorder (ADHD) during young age. Front Behav Neurosci. 2020;14:27. 32174817 10.3389/fnbeh.2020.00027 PMC7054716

[47] Bouchatta O, Manouze H, Bouali-Benazzouz R, Kerekes N, Ba-M’hamed S, Fossat P, et al. Neonatal 6-OHDA lesion model in mouse induces attention-deficit/hyperactivity disorder (ADHD)-like behaviour. Sci Rep. 2018;8(1):15349. 30337626 10.1038/s41598-018-33778-0 PMC6193955

[48] Luthman J, Fredriksson A, Lewander T, Jonsson G, Archer T. Effects of d-amphetamine and methylphenidate on hyperactivity produced by neonatal 6-hydroxydopamine treatment. Psychopharmacology. 1989;99(4):550–7. 2594922 10.1007/BF00589907

[49] Shaywitz BA, Klopper JH, Yager RD, Gordon JW. Paradoxical response to amphetamine in developing rats treated with 6-hydroxydopamine. Nature. 1976;261(5556):153–5. 944861 10.1038/261153a0

[50] Zhang K, Tarazi FI, Davids E, Baldessarini RJ. Plasticity of dopamine d4 receptors in rat forebrain: temporal association with motor hyperactivity following neonatal 6-hydroxydopamine lesioning. Neuropsychopharmacology. 2002;26(5):625–33. 11927187 10.1016/S0893-133X(01)00404-3

[51] Erinoff L, MacPhail RC, Heller A, Seiden LS. Age-dependent effects of 6-hydroxydopamine on locomotor activity in the rat. Brain Res. 1979;164:195–205. 427556 10.1016/0006-8993(79)90015-5

[52] Carreon-Trujillo S, Corona JC. No effects of decanoic acid on locomotor activity and antioxidant defences in an experimental animal model of Attention-Deficit/Hyperactivity disorder. J Integr Neurosci. 2024;23(2):39. 38419446 10.31083/j.jin2302039

[53] Vazquez-Gonzalez D, Corona JC. Pioglitazone enhances brain mitochondrial biogenesis and phase II detoxification capacity in neonatal rats with 6-OHDA-induced unilateral striatal lesions. Front Neurosci. 2023;17:1186520. 37575308 10.3389/fnins.2023.1186520 PMC10416244

[54] Archer T, Danysz W, Fredriksson A, Jonsson G, Luthman J, Sundstrom E, et al. Neonatal 6-hydroxydopamine-induced dopamine depletions: motor activity and performance in maze learning. Pharmacol Biochem Behav. 1988;31(2):357–64. 3149743 10.1016/0091-3057(88)90358-9

[55] LaHoste GJ, Swanson JM, Wigal SB, Glabe C, Wigal T, King N, et al. Dopamine D4 receptor gene polymorphism is associated with attention deficit hyperactivity disorder. Mol Psychiatry. 1996;1(2):121–4. 9118321

[56] Dougherty DD, Bonab AA, Spencer TJ, Rauch SL, Madras BK, Fischman AJ. Dopamine transporter density in patients with attention deficit hyperactivity disorder. Lancet. 1999;354(9196):2132–3. 10609822 10.1016/S0140-6736(99)04030-1

[57] Davids E, Zhang K, Kula NS, Tarazi FI, Baldessarini RJ. Effects of norepinephrine and serotonin transporter inhibitors on hyperactivity induced by neonatal 6-hydroxydopamine lesioning in rats. J Pharmacol Exp Ther. 2002;301(3):1097–102. 12023542 10.1124/jpet.301.3.1097

[58] Heffner TG, Seiden LS. Possible involvement of serotonergic neurons in the reduction of locomotor hyperactivity caused by amphetamine in neonatal rats depleted of brain dopamine. Brain Res. 1982;244(1):81–90. 6288184 10.1016/0006-8993(82)90906-4

[59] Moran-Gates T, Zhang K, Baldessarini RJ, Tarazi FI. Atomoxetine blocks motor hyperactivity in neonatal 6-hydroxydopamine-lesioned rats: implications for treatment of attention-deficit hyperactivity disorder. Int J Neuropsychopharmacol. 2005;8(3):439–44. 15817135 10.1017/S1461145705005249

[60] Teicher MH, Andersen SL, Campbell A, Gelbard HA, Baldessarini RJ. Progressive accumbens degeneration after neonatal striatal 6-hydroxydopamine in rats. Neurosci Lett. 1998;247(2–3):99–102. 9655602 10.1016/s0304-3940(98)00281-x

[61] Caballero M, Nunez F, Ahern S, Cuffi ML, Carbonell L, Sanchez S, et al. Caffeine improves attention deficit in neonatal 6-OHDA lesioned rats, an animal model of attention deficit hyperactivity disorder (ADHD). Neurosci Lett. 2011;494(1):44–8. 21362462 10.1016/j.neulet.2011.02.050

[62] Zhu J, Zhang X, Xu Y, Spencer TJ, Biederman J, Bhide PG. Prenatal nicotine exposure mouse model showing hyperactivity, reduced cingulate cortex volume, reduced dopamine turnover, and responsiveness to oral methylphenidate treatment. J Neurosci. 2012;32(27):9410–8. 22764249 10.1523/JNEUROSCI.1041-12.2012 PMC3417040

[63] Bryden DW, Burton AC, Barnett BR, Cohen VJ, Hearn TN, Jones EA, et al. Prenatal nicotine exposure impairs executive control signals in medial prefrontal cortex. Neuropsychopharmacology. 2016;41(3):716–25. 26189451 10.1038/npp.2015.197 PMC4707818

[64] Fahlke C, Hansen S. Alcohol responsiveness, hyperreactivity, and motor restlessness in an animal model for attention-deficit hyperactivity disorder. Psychopharmacology. 1999;146(1):1–9. 10485958 10.1007/s002130051081

[65] Wang R, Martin CD, Lei AL, Hausknecht KA, Ishiwari K, Richards JB, et al. Prenatal ethanol exposure leads to attention deficits in both male and female rats. Front Neurosci. 2020;14:12. 32038156 10.3389/fnins.2020.00012 PMC6992663

[66] Silbergeld EK, Goldberg AM. Lead-induced behavioral dysfunction: an animal model of hyperactivity. Exp Neurol. 1974;42(1):146–57. 4856900 10.1016/0014-4886(74)90013-2

[67] Kern CH, Smith DR. Preweaning Mn exposure leads to prolonged astrocyte activation and lasting effects on the dopaminergic system in adult male rats. Synapse. 2011;65(6):532–44. 20963817 10.1002/syn.20873 PMC3070959

[68] Dell’Anna ME, Calzolari S, Molinari M, Iuvone L, Calimici R. Neonatal anoxia induces transitory hyperactivity, permanent spatial memory deficits and CA1 cell density reduction in developing rats. Behav Brain Res. 1991;45(2):125–34. 1789921 10.1016/s0166-4328(05)80078-6

[69] Cada AM, Gray EP, Ferguson SA. Minimal behavioral effects from developmental cerebellar stunting in young rats induced by postnatal treatment with alpha-difluoromethylornithine. Neurotoxicol Teratol. 2000;22(3):415–20. 10840185 10.1016/s0892-0362(99)00085-9

[70] Ferguson SA, Cada AM, Gray EP, Paule MG. No alterations in the performance of two interval timing operant tasks after alpha-difluoromethylornithine (DFMO)-induced cerebellar stunting. Behav Brain Res. 2001;126(1–2):135–46. 11704259 10.1016/s0166-4328(01)00259-5

[71] Son GH, Chung S, Geum D, Kang SS, Choi WS, Kim K, et al. Hyperactivity and alteration of the midbrain dopaminergic system in maternally stressed male mice offspring. Biochem Biophys Res Commun. 2007;352(3):823–9. 17150178 10.1016/j.bbrc.2006.11.104

[72] Jeon SC, Kim HJ, Ko EA, Jung SC. Prenatal exposure to high cortisol induces ADHD-like behaviors with delay in spatial cognitive functions during the post-weaning period in rats. Exp Neurobiol. 2021;30(1):87–100. 33632985 10.5607/en20057 PMC7926048

[73] Godleski S, Shisler S, Colton K, Leising M. Prenatal tobacco exposure and behavioral disorders in children and adolescents: systematic review and meta-analysis. Pediatr Rep. 2024;16(3):736–52. 39311325 10.3390/pediatric16030062 PMC11417955

[74] Jansone K, Eichler A, Fasching PA, Kornhuber J, Kaiser A, Millenet S, et al. Association of maternal smoking during pregnancy with neurophysiological and ADHD-related outcomes in school-aged children. Int J Environ Res Public Health. 2023;20(6):4716. 36981624 10.3390/ijerph20064716 PMC10048892

[75] Schneider T, Bizarro L, Asherson PJ, Stolerman IP. Hyperactivity, increased nicotine consumption and impaired performance in the five-choice serial reaction time task in adolescent rats prenatally exposed to nicotine. Psychopharmacology. 2012;223(4):401–15. 22562524 10.1007/s00213-012-2728-7 PMC4765091

[76] Zhu J, Lee KP, Spencer TJ, Biederman J, Bhide PG. Transgenerational transmission of hyperactivity in a mouse model of ADHD. J Neurosci. 2014;34(8):2768–73. 24553919 10.1523/JNEUROSCI.4402-13.2014 PMC3931498

[77] Zhang L, Spencer TJ, Biederman J, Bhide PG. Attention and working memory deficits in a perinatal nicotine exposure mouse model. PLoS ONE. 2018;13(5):e0198064. 29795664 10.1371/journal.pone.0198064 PMC5967717

[78] Jm M, Fj J, Gm M, As K, Fp M. Prenatal alcohol exposure and risk of attention deficit hyperactivity disorder in offspring: A retrospective analysis of the millennium cohort study. J Affect Disord. 2020;269:94–100. 32250868 10.1016/j.jad.2020.03.027

[79] Pagnin D, Zamboni Grecco ML, Furtado EF. Prenatal alcohol use as a risk for attention-deficit/hyperactivity disorder. Eur Arch Psychiatry Clin Neurosci. 2019;269(6):681–7. 30353263 10.1007/s00406-018-0946-7

[80] Hausknecht KA, Acheson A, Farrar AM, Kieres AK, Shen RY, Richards JB, et al. Prenatal alcohol exposure causes attention deficits in male rats. Behav Neurosci. 2005;119(1):302–10. 15727534 10.1037/0735-7044.119.1.302

[81] Aghaie CI, Hausknecht KA, Wang R, Dezfuli PH, Haj-Dahmane S, Kane CJM, et al. Prenatal ethanol exposure and postnatal environmental intervention alter dopaminergic neuron and microglia morphology in the ventral tegmental area during adulthood. Alcohol Clin Exp Res. 2020;44(2):435–44. 31872887 10.1111/acer.14275 PMC7153307

[82] Shen RY, Choong KC. Different adaptations in ventral tegmental area dopamine neurons in control and ethanol exposed rats after methylphenidate treatment. Biol Psychiatry. 2006;59(7):635–42. 16199009 10.1016/j.biopsych.2005.08.021

[83] Shen RY, Choong KC, Thompson AC. Long-term reduction in ventral tegmental area dopamine neuron population activity following repeated stimulant or ethanol treatment. Biol Psychiatry. 2007;61(1):93–100. 16697354 10.1016/j.biopsych.2006.03.018

[84] Xu C, Shen RY. Amphetamine normalizes the electrical activity of dopamine neurons in the ventral tegmental area following prenatal ethanol exposure. J Pharmacol Exp Ther. 2001;297(2):746–52. 11303066

[85] Lee MJ, Chou MC, Chou WJ, Huang CW, Kuo HC, Lee SY, et al. Heavy metals’ effect on susceptibility to attention-deficit/hyperactivity disorder: implication of lead, cadmium, and antimony. Int J Environ Res Public Health. 2018;15(6):1221. 29890770 10.3390/ijerph15061221 PMC6025252

[86] Silbergeld EK, Goldberg AM. Hyperactivity. A lead-induced behavior disorder. Environ Health Perspect. 1974;7:227–32. 4857498 10.1289/ehp.747227 PMC1475143

[87] Silbergeld EK, Goldberg AM. Pharmacological and neurochemical investigations of lead-induced hyperactivity. Neuropharmacology. 1975;14(5–6):431–44. 1171389 10.1016/0028-3908(75)90026-x

[88] Memo M, Lucchi L, Spano PF, Trabucchi M. Dose-dependent and reversible effects of lead on rat dopaminergic system. Life Sci. 1981;28(7):795–9. 7231055 10.1016/0024-3205(81)90163-6

[89] Wong KL, Klaassen CD. Neurotoxic effects of cadmium in young rats. Toxicol Appl Pharmacol. 1982;63(3):330–7. 7101297 10.1016/0041-008x(82)90261-7

[90] Kern CH, Stanwood GD, Smith DR. Preweaning manganese exposure causes hyperactivity, disinhibition, and spatial learning and memory deficits associated with altered dopamine receptor and transporter levels. Synapse. 2010;64(5):363–78. 20029834 10.1002/syn.20736 PMC2840192

[91] Smith TF, Schmidt-Kastner R, McGeary JE, Kaczorowski JA, Knopik VS. Pre- and perinatal ischemia-hypoxia, the ischemia-hypoxia response pathway, and ADHD risk. Behav Genet. 2016;46(3):467–77. 26920003 10.1007/s10519-016-9784-4

[92] Speiser Z, Korczyn AD, Teplitzky I, Gitter S. Hyperactivity in rats following postnatal anoxia. Behav Brain Res. 1983;7(3):379–82. 6682332 10.1016/0166-4328(83)90028-1

[93] Speiser Z, Amitzi-Sonder J, Gitter S, Cohen S. Behavioral differences in the developing rat following postnatal anoxia or postnatally injected AF-64A, a cholinergic neurotoxin. Behav Brain Res. 1988;30(1):89–94. 3166708 10.1016/0166-4328(88)90010-1

[94] Shimomura C, Ohta H. Behavioral abnormalities and seizure susceptibility in rat after neonatal anoxia. Brain Dev. 1988;10(3):160–3. 3407852 10.1016/s0387-7604(88)80020-2

[95] Decker MJ, Hue GE, Caudle WM, Miller GW, Keating GL, Rye DB. Episodic neonatal hypoxia evokes executive dysfunction and regionally specific alterations in markers of dopamine signaling. Neuroscience. 2003;117(2):417–25. 12614682 10.1016/s0306-4522(02)00805-9

[96] Row BW, Kheirandish L, Neville JJ, Gozal D. Impaired spatial learning and hyperactivity in developing rats exposed to intermittent hypoxia. Pediatr Res. 2002;52(3):449–53. 12193683 10.1203/00006450-200209000-00024

[97] Miguel PM, Schuch CP, Rojas JJ, Carletti JV, Deckmann I, Martinato LH, et al. Neonatal hypoxia-ischemia induces attention-deficit hyperactivity disorder-like behavior in rats. Behav Neurosci. 2015;129(3):309–20. 26030430 10.1037/bne0000063

[98] Gramatte T, Schmidt J. The effect of early postnatal hypoxia on the development of locomotor activity in rats. Biomed Biochim Acta. 1986;45(4):523–9. 3707565

[99] Dell’Anna ME, Luthman J, Lindqvist E, Olson L. Development of monoamine systems after neonatal anoxia in rats. Brain Res Bull. 1993;32(2):159–70. 8348340 10.1016/0361-9230(93)90070-r

[100] Berquin PC, Giedd JN, Jacobsen LK, Hamburger SD, Krain AL, Rapoport JL, et al. Cerebellum in attention-deficit hyperactivity disorder: a morphometric MRI study. Neurology. 1998;50(4):1087–93. 9566399 10.1212/wnl.50.4.1087

[101] Mostofsky SH, Reiss AL, Lockhart P, Denckla MB. Evaluation of cerebellar size in attention-deficit hyperactivity disorder. J Child Neurol. 1998;13(9):434–9. 9733289 10.1177/088307389801300904

[102] Bruchhage MMK, Bucci MP, Becker EBE. Cerebellar involvement in autism and ADHD. Handb Clin Neurol. 2018;155:61–72. 29891077 10.1016/B978-0-444-64189-2.00004-4

[103] Ferguson SA, Paule MG, Holson RR. Functional effects of methylazoxymethanol-induced cerebellar hypoplasia in rats. Neurotoxicol Teratol. 1996;18(5):529–37. 8888017 10.1016/0892-0362(96)00083-9

[104] Ferguson SA, Holson RR. Neonatal dexamethasone on day 7 causes mild hyperactivity and cerebellar stunting. Neurotoxicol Teratol. 1999;21(1):71–6. 10023803 10.1016/s0892-0362(98)00029-4

[105] Ronald A, Pennell CE, Whitehouse AJ. Prenatal maternal stress associated with ADHD and autistic traits in early childhood. Front Psychol. 2011;1:223. 21833278 10.3389/fpsyg.2010.00223 PMC3153828

[106] Tong P, Kong G, Shi Y, Dong L, Bo P. Maternal stress during pregnancy leads to ADHD like behavior in offspring mice and its mechanism. Chin J Behav Med Brain Sci. 2021;12:200–5.

[107] Kim HJ, Ko EA, Kwon OB, Jung SC. Prenatal treatment with corticosterone via maternal injection induces learning and memory impairments via delaying postsynaptic development in hippocampal CA1 neurons of rats. J Neurosci Res. 2024;102(4):e25323. 38553948 10.1002/jnr.25323

[108] Okamoto K, Aoki K. Development of a strain of spontaneously hypertensive rats. Jpn Circ J. 1963;27:282–93. 13939773 10.1253/jcj.27.282

[109] Moser MB, Moser EI, Wultz B, Sagvolden T. Component analyses differentiate between exploratory behaviour of spontaneously hypertensive rats and Wistar Kyoto rats in a two-compartment free-exploration open field. Scand J Psychol. 1988;29(3–4):200–6. 3232042 10.1111/j.1467-9450.1988.tb00792.x

[110] Wyss JM, Fisk G, van Groen T. Impaired learning and memory in mature spontaneously hypertensive rats. Brain Res. 1992;592(1–2):135–40. 1450905 10.1016/0006-8993(92)91668-5

[111] Sagvolden T. Behavioral validation of the spontaneously hypertensive rat (SHR) as an animal model of attention-deficit/hyperactivity disorder (ADHD). Neurosci Biobehav Rev. 2000;24(1):31–9. 10654658 10.1016/s0149-7634(99)00058-5

[112] Johansen EB, Killeen PR, Sagvolden T. Behavioral variability, elimination of responses, and delay-of-reinforcement gradients in SHR and WKY rats. Behav Brain Funct. 2007;3:60. 18028539 10.1186/1744-9081-3-60 PMC2219961

[113] Boix F, Qiao SW, Kolpus T, Sagvolden T. Chronic L-deprenyl treatment alters brain monoamine levels and reduces impulsiveness in an animal model of Attention-Deficit/Hyperactivity disorder. Behav Brain Res. 1998;94(1):153–62. 9708846 10.1016/s0166-4328(97)00176-9

[114] Sagvolden T, Johansen EB, Woien G, Walaas SI, Storm-Mathisen J, Bergersen LH, et al. The spontaneously hypertensive rat model of ADHD–the importance of selecting the appropriate reference strain. Neuropharmacology. 2009;57(7–8):619–26. 19698722 10.1016/j.neuropharm.2009.08.004 PMC2783904

[115] Gungor Aydin A, Adiguzel E. The mesocortical dopaminergic system cannot explain hyperactivity in an animal model of attention deficit hyperactivity disorder (ADHD)- spontaneously hypertensive rats (SHR). Lab Anim Res. 2023;39(1):20. 37710339 10.1186/s42826-023-00172-5 PMC10500870

[116] Yamashita M, Sakakibara Y, Hall FS, Numachi Y, Yoshida S, Kobayashi H, et al. Impaired cliff avoidance reaction in dopamine transporter knockout mice. Psychopharmacology. 2013;227(4):741–9. 23397052 10.1007/s00213-013-3009-9

[117] Li B, Arime Y, Hall FS, Uhl GR, Sora I. Impaired spatial working memory and decreased frontal cortex BDNF protein level in dopamine transporter knockout mice. Eur J Pharmacol. 2010;628(1–3):104–7. 19932884 10.1016/j.ejphar.2009.11.036 PMC3724416

[118] Raber J, Mehta PP, Kreifeldt M, Parsons LH, Weiss F, Bloom FE, et al. Coloboma hyperactive mutant mice exhibit regional and transmitter-specific deficits in neurotransmission. J Neurochem. 1997;68(1):176–86. 8978724 10.1046/j.1471-4159.1997.68010176.x

[119] Bruno KJ, Freet CS, Twining RC, Egami K, Grigson PS, Hess EJ. Abnormal latent inhibition and impulsivity in Coloboma mice, a model of ADHD. Neurobiol Dis. 2007;25(1):206–16. 17064920 10.1016/j.nbd.2006.09.009 PMC1761697

[120] Hess EJ, Jinnah HA, Kozak CA, Wilson MC. Spontaneous locomotor hyperactivity in a mouse mutant with a deletion including the snap gene on chromosome 2. J Neurosci. 1992;12(7):2865–74. 1613559 10.1523/JNEUROSCI.12-07-02865.1992 PMC6575838

[121] Siesser WB, Zhao J, Miller LR, Cheng SY, McDonald MP. Transgenic mice expressing a human mutant beta1 thyroid receptor are hyperactive, impulsive, and inattentive. Genes Brain Behav. 2006;5(3):282–97. 16594981 10.1111/j.1601-183X.2005.00161.x

[122] McDonald MP, Wong R, Goldstein G, Weintraub B, Cheng SY, Crawley JN. Hyperactivity and learning deficits in transgenic mice bearing a human mutant thyroid hormone beta1 receptor gene. Learn Mem. 1998;5(4–5):289–301. 10454355 PMC311272

[123] Porter AJ, Pillidge K, Tsai YC, Dudley JA, Hunt SP, Peirson SN, et al. A lack of functional nk1 receptors explains most, but not all, abnormal behaviours of NK1R-/- mice(1). Genes Brain Behav. 2015;14(2):189–99. 25558794 10.1111/gbb.12195 PMC4415486

[124] Yan TC, Hunt SP, Stanford SC. Behavioural and neurochemical abnormalities in mice lacking functional tachykinin-1 (NK1) receptors: a model of attention deficit hyperactivity disorder. Neuropharmacology. 2009;57(7–8):627–35. 19748515 10.1016/j.neuropharm.2009.08.021

[125] Gainetdinov RR, Jones SR, Caron MG. Functional hyperdopaminergia in dopamine transporter knock-out mice. Biol Psychiatry. 1999;46(3):303–11. 10435196 10.1016/s0006-3223(99)00122-5

[126] Gainetdinov RR, Caron MG. Genetics of childhood disorders: XXIV. ADHD, part 8: hyperdopaminergic mice as an animal model of ADHD. J Am Acad Child Adolesc Psychiatry. 2001;40(3):380–2. 11288782 10.1097/00004583-200103000-00020

[127] Giros B, Jaber M, Jones SR, Wightman RM, Caron MG. Hyperlocomotion and indifference to cocaine and amphetamine in mice lacking the dopamine transporter. Nature. 1996;379(6566):606–12. 8628395 10.1038/379606a0

[128] Jones SR, Gainetdinov RR, Jaber M, Giros B, Wightman RM, Caron MG. Profound neuronal plasticity in response to inactivation of the dopamine transporter. Proc Natl Acad Sci U S A. 1998;95(7):4029–34. 9520487 10.1073/pnas.95.7.4029 PMC19957

[129] Cheon KA, Ryu YH, Kim YK, Namkoong K, Kim CH, Lee JD. Dopamine transporter density in the basal ganglia assessed with [123i]IPT SPET in children with attention deficit hyperactivity disorder. Eur J Nucl Med Mol Imaging. 2003;30(2):306–11. 12552351 10.1007/s00259-002-1047-3

[130] Krause KH, Dresel SH, Krause J, Kung HF, Tatsch K. Increased striatal dopamine transporter in adult patients with attention deficit hyperactivity disorder: effects of methylphenidate as measured by single photon emission computed tomography. Neurosci Lett. 2000;285(2):107–10. 10793238 10.1016/s0304-3940(00)01040-5

[131] Hesse S, Ballaschke O, Barthel H, Sabri O. Dopamine transporter imaging in adult patients with attention-deficit/hyperactivity disorder. Psychiatry Res. 2009;171(2):120–8. 19176281 10.1016/j.pscychresns.2008.01.002

[132] Wilson MC. Coloboma mouse mutant as an animal model of hyperkinesis and attention deficit hyperactivity disorder. Neurosci Biobehav Rev. 2000;24(1):51–7. 10654661 10.1016/s0149-7634(99)00064-0

[133] Hess EJ, Collins KA, Wilson MC. Mouse model of hyperkinesis implicates SNAP-25 in behavioral regulation. J Neurosci. 1996;16(9):3104–11. 8622140 10.1523/JNEUROSCI.16-09-03104.1996 PMC6579059

[134] Weiss RE, Refetoff S. Resistance to thyroid hormone. Rev Endocr Metab Disord. 2000;1(1–2):97–108. 11704998 10.1023/a:1010072605757

[135] Modesto T, Tiemeier H, Peeters RP, Jaddoe VW, Hofman A, Verhulst FC, et al. Maternal mild thyroid hormone insufficiency in early pregnancy and Attention-Deficit/Hyperactivity disorder symptoms in children. JAMA Pediatr. 2015;169(9):838–45. 26146876 10.1001/jamapediatrics.2015.0498

[136] Brucker-Davis F, Skarulis MC, Grace MB, Benichou J, Hauser P, Wiggs E, et al. Genetic and clinical features of 42 kindreds with resistance to thyroid hormone. The National Institutes of health prospective study. Ann Intern Med. 1995;123(8):572–83. 7677297 10.7326/0003-4819-123-8-199510150-00002

[137] Siesser WB, Cheng SY, McDonald MP. Hyperactivity, impaired learning on a vigilance task, and a differential response to methylphenidate in the TRbetaPV knock-in mouse. Psychopharmacology. 2005;181(4):653–63. 15983791 10.1007/s00213-005-0024-5

[138] Thompson CC, Potter GB. Thyroid hormone action in neural development. Cereb Cortex. 2000;10(10):939–45. 11007544 10.1093/cercor/10.10.939

[139] Sharp SI, McQuillin A, Marks M, Hunt SP, Stanford SC, Lydall GJ, et al. Genetic association of the Tachykinin receptor 1 TARC1 gene in bipolar disorder, attention deficit hyperactivity disorder, and the alcohol dependence syndrome. Am J Med Genet B Neuropsychiatr Genet. 2014;165B(4):373–80. 24817687 10.1002/ajmg.b.32241 PMC4278563

[140] Yan TC, McQuillin A, Thapar A, Asherson P, Hunt SP, Stanford SC, et al. Nk1 (TARC1) receptor gene ‘knockout’ mouse phenotype predicts genetic association with ADHD. J Psychopharmacol. 2010;24(1):27–38. 19204064 10.1177/0269881108100255 PMC3943619

[141] Fisher AS, Stewart RJ, Yan T, Hunt SP, Stanford SC. Disruption of noradrenergic transmission and the behavioural response to a novel environment in NK1R-/- mice. Eur J Neurosci. 2007;25(4):1195–204. 17331215 10.1111/j.1460-9568.2007.05369.x

[142] Herpfer I, Hunt SP, Stanford SC. A comparison of neurokinin 1 receptor knock-out (NK1-/-) and wildtype mice: exploratory behaviour and extracellular noradrenaline concentration in the cerebral cortex of anaesthetised subjects. Neuropharmacology. 2005;48(5):706–19. 15814105 10.1016/j.neuropharm.2004.12.016

[143] Dudley JA, Weir RK, Yan TC, Grabowska EM, Grimme AJ, Amini S, et al. Antagonism of L-type Ca(v) channels with Nifedipine differentially affects performance of wildtype and NK1R-/- mice in the 5-choice serial reaction-time task. Neuropharmacology. 2013;64:329–36. 22884624 10.1016/j.neuropharm.2012.06.056

[144] Pillidge K, Porter AJ, Young JW, Stanford SC. Perseveration by NK1R-/- (‘knockout’) mice is blunted by doses of methylphenidate that affect neither other aspects of their cognitive performance nor the behaviour of wild-type mice in the 5-choice continuous performance test. J Psychopharmacol. 2016;30(9):837–47. 27097734 10.1177/0269881116642541 PMC4994704

[145] Yan TC, Dudley JA, Weir RK, Grabowska EM, Pena-Oliver Y, Ripley TL, et al. Performance deficits of NK1 receptor knockout mice in the 5-choice serial reaction-time task: effects of D-amphetamine, stress and time of day. PLoS ONE. 2011;6(3):e17586. 21408181 10.1371/journal.pone.0017586 PMC3049786

[146] Pillidge K, Porter AJ, Vasili T, Heal DJ, Stanford SC. Atomoxetine reduces hyperactive/impulsive behaviours in neurokinin-1 receptor ‘knockout’ mice. Pharmacol Biochem Behav. 2014;127:56–61. 25450119 10.1016/j.pbb.2014.10.008 PMC4258612

[147] Froger N, Gardier AM, Moratalla R, Alberti I, Lena I, Boni C, et al. 5-hydroxytryptamine (5-HT)1A autoreceptor adaptive changes in substance p (neurokinin 1) receptor knock-out mice mimic antidepressant-induced desensitization. J Neurosci. 2001;21(20):8188–97. 11588191 10.1523/JNEUROSCI.21-20-08188.2001 PMC6763873

[148] de Jong W, Linthorst AC, Versteeg HG. The nigrostriatal dopamine system and the development of hypertension in the spontaneously hypertensive rat. Arch Mal Coeur Vaiss. 1995;88(8):1193–6. 8572872

